# Application of an Ecotoxicological Battery Test to the Paddy Field Soils of the Albufera Natural Park

**DOI:** 10.3390/toxics10070375

**Published:** 2022-07-05

**Authors:** Oscar Andreu-Sánchez, Jesús Moratalla-López, José Antonio Rodríguez-Martín, Luis Roca-Pérez

**Affiliations:** 1 Departamento de Biología Celular, Biología Funcional y Antropología Física, Laboratorio de Ecotoxicología y Calidad Ambiental (LEyCA), Universitat de València, 46100 Burjassot, Spain; 2 Departamento de Biología Vegetal, Facultad de Farmacia, Universitat de València, 46100 Burjassot, Spain; moloje@uv.es (J.M.-L.); luis.roca@uv.es (L.R.-P.); 3 Departamento de Medio Ambiente, Instituto Nacional de Investigación y Tecnología Agraria y Alimentaria (INIA-CSIC), Carretera de La Coruña, km 7.5, 28040 Madrid, Spain; rmartin@inia.es

**Keywords:** heavy metals, ecotoxicity, bioassays, Albufera Natural Park, risk assessment

## Abstract

Albufera Natural Park (ANP) (Valencia, Spain) is one of the most important wetland areas of the Mediterranean coast subject to high anthropogenic pressure, on whose soils a battery of bioassays has never been applied to evaluate the ecotoxicological risk. The present study determined available and water-soluble heavy metal content in four paddy soils used in the ANP, and the ecotoxicological effect on these soils was evaluated by performing the bioassays regulated in Spanish Royal Decree 9/2005. Soil properties and extractable Co, Cr, Cu, Ni, Pb and Zn (EDTA pH = 7) were analyzed in soils. These elements and macro- and micronutrients were also assessed in soil leachate. A test battery covering the following was needed: acute toxicity test in *Eisenia foetida* (OECD TG 207); mineralization tests of nitrogen (OECD TG 2016) and carbon (OECD TG 217); growth inhibition test in *Raphidocelis subcapitata* (OECD TG 201); mobility inhibition test in *Daphnia magna* (OECD TG 202). The soils found in the most anthropized areas to the north of the ANP (Massanassa and Alfafar) demonstrated a higher concentration of available heavy metals than in the southern ones (Sueca and Sollana). The aqueous leachate of the studied soils contained very low concentrations, which would be related to soil properties. Despite the high concentration of available potentially toxic elements (PTEs) in the Massanassa and Alfafar soils, the studied soils showed no toxicity during the performed battery bioassays. Therefore, soils can be considered non-toxic despite the obtained PTEs available concentration.

## 1. Introduction

Potentially toxic (trace) elements (PTEs) have attracted much attention for years given their persistence and high risk to ecosystems. Toxic elements that potentially cause pollution result from rock alteration, agriculture, industry and other anthropogenic activities, and their mobility and bioavailability depend on the form in which they are found in the environmental compartment [1,2,3]. Soils act as a sink for heavy metals and metalloids from anthropic activities, like fertilization, application of pesticides and organic amendments (wastewater sludge, manure, etc.), mining, military and urban actions, and industrial waste spillages. Soil parameters, such as clay fraction, organic matter content, oxidation state, pH, redox potential, aeration and the presence of specific organisms, play fundamental roles in the bioavailability of essential trace metals [2,4,5,6,7]. According to their bioavailability, the toxicity of heavy metals and metalloids is higher or lower for organisms, and bioassays establish the ecotoxicity of these elements in soil [4,8,9]. EDTA (Ethylenediaminetetraacetic acid) is normally employed as an extractant to assess the bioavailable fraction of heavy metals and metalloids in soil [7,8,10,11,12] by allowing the elements united in the organic fraction and linked with sulfurs to be extracted, which correspond to the fraction available for plants [5,7].

The Albufera Natural Park (ANP) is a protected Mediterranean wetland affected by human activities. This park includes special protected zones for birds, and is on the list of international wetlands (The Ramsar Convention). Toxicity tests for predicting environmental effects are necessary in environmental risk assessments. To the best of our knowledge, no environmental risk assessment integrates both ecotoxicological (aquatic and terrestrial) tests and occurrence of trace metals in paddy soils from the ANP. In Spain, the use of ecotoxicity tests to assess potentially polluted soil is regulated by Spanish Royal Decree (RD) 9/2005 [13], which outlines potentially polluting activities, and establishes the criteria and standards by which soil can be declared as polluted. The methodology for demonstrating soil degradation as a result of the presence of a pollutant is based on a battery of different OECD (Organization for Economic Co-operation and Development) Technical Guidelines (TG), e.g., OECD TG 207 (earthworm mortality), OECD TG 208 (germination and root elongation), OECD TG 216 (soil N mineralization) and OECD TG 217 (soil C mineralization), conducted with problem soil, and OECD TG 201 (growth inhibition in algae), OECD TG 202 (*Daphnia magna* immobilization) and OECD TG 203 (fish mortality) conducted with aqueous extract (leachate), generated according to Standard DIN 38414-S4 [14].

Several ecotoxicity studies report different results depending on test and soil properties [15,16]. Increasing heavy metals and metalloids contents can have a negative effect on the C mineralization rate, as determined by respirometry techniques, and also on N transformation [2,17,18]. However, some studies have applied heavy metal in soils with high organic matter contents and found no toxic effect after adding these elements [19]. Obviously, there are also potential associated effects at high nutrient levels. After performing several bioassays, the *Eisenia foetida* mortality test was the least sensitive, while the most sensitive one was the test that employed *Daphnia magna* immobilization for soils polluted by trace elements from mining activities [9]. Thus bioassays like pot bioassay, soil plate bioassay and Zucconi test, which were performed in the paddy field soils in the ANP that were polluted by PTEs, were poorly sensitive to pollution by these elements, whereas the plate bioassay was sensitive in the soils with a higher content of the tested pollutants. However, the possible toxicity effect could be related to the salinity of these soils [20]. Sewage effluents are considered by some authors to be sources of emerging pollutants and personal care products, among others [21,22]. Adverse biological effects come into play because some authors have studied the reuse of polluted sediments and polluted ravine waters in the paddy fields surrounding the ANP [23,24].

This study aimed to assess the pollution by heavy metals of the paddy field soils in the ANP by performing the ecotoxicological bioassays set by Spanish RD 9/2005 [13]. To do so, soil properties and heavy metals content (available and extractable in water) were determined in four soils used for growing rice in the ANP. Acute toxicity testing was also carried out in *Eisenia foetida* (OECD TG 207), as were N mineralization (OECD TG 216) and C mineralization (OECD TG 217) tests, a growth inhibition test in *Raphidocelis subcapitata* (OECD TG 201) and a mobility inhibition test in *Daphnia magna* (OECD TG 202).

## 2. Materials and Methods

### 2.1. Study Area and Sampling

The ANP is a wetland located 12 km south of the city of Valencia (east Spain, 39°20′ N, 0°21′ W) and is formed by the Albufera Lagoon, a Mediterranean coastal oligohaline lagoon that covers 23.2 km^2^, which represents approximately 11% of the wetland area [25]. The zone includes part of the main rivers, irrigation channels and inflows and outflows, and is surrounded mostly by paddy fields, but also by some industries and companies. For this reason, the area is continuously affected by the environmental effects of human activities and water uses, related mainly to agriculture and urban/industrial sewage discharges [26]. This complex irrigation system connects many canals to the sewerage infrastructures of the surrounding municipalities, and receives industrial and urban discharges.

**Figure 1:**
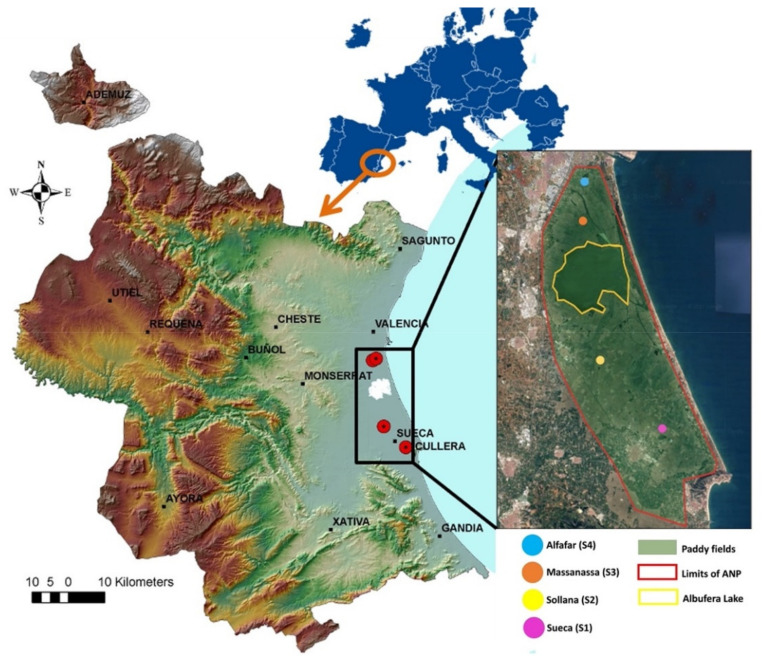
Map of Valencia showing the soil samples of paddy fields in Albufera Natural Park.

Soil samples were taken from the different paddy fields in the ANP to perform the bioassays (Figure 1). The samples taken in the towns of Massanassa (S3) and Alfafar (S4) are characterized by being close to a highly anthropized area (industrial estates, town centers, excess fertilizer and pesticide uses, and waste and sewage are dumped into irrigation channels and the lagoon). The towns of Sueca (S1) and Sollana (S2) are located in less anthropized places [27,28,29,30,31]. Soil samples were collected in October 2020 from a depth of 0–20 cm. They were crushed, homogenized, sieved through a 2 mm mesh and subjected to physico-chemical characterization. Soils were classified as gleyic-calcareous Fluvisol according to Boluda et al. [20].

### 2.2. Soil Analyses

Soil pH was measured in a 1:5 *v*/*v* (soil/distilled water) extract [32]. Soil organic matter (SOM) was analyzed by the Walkley–Black method [33]. Particle size was determined by sedimentation [34]. Electrical conductivity (EC) was measured in the 1:5 (*w*/*v*) extract [35]. Carbonate content was established in a Bernad calcimeter [36]. Cation exchange capacity (CEC) was determined in acetate ammonic extract according to Boluda et al. [20]. Nitrogen (N) was established in an EA 1110 (CE Instruments) elemental analyzer [37]. To determine the available fraction of heavy metals, EDTA extraction was used as described by Ramos-Miras et al. [7]. Concentrations of elements were determined by atomic absorption spectrometry (AAS) and inductively coupled plasma-optical emission spectrometry (ICP-OES). The detection limits of elements in mg/L for AAS were 0.036 for Cr, 0.005 for Cu, 0.032 for Co, 0.053 for Ni, 0.04 for Pb and 0.034 for Zn. Accuracy (expressed as %) was 19.0.1 for Cr, 11.54 for Cu, 8.01 for Co, 10.15 for Ni, 13.03 for Pb and 10.50 for Zn. The Zn equivalent (ZnEq) was determined as described by Gil et al. [38].

### 2.3. Terrestrial Ecotoxicity Tests

This study focused on the OECD TGs battery tests based on Spanish RD 9/2005 [13]. Direct exposure bioassays were conducted in soil samples and indirect ecotoxicity tests were performed in soil leachate. Direct exposure toxicity bioassays were carried out using whole soils. When dilution was needed, test soils were mixed with artificial soil (70% quartz sand (granulometry: 80–120 μm), 20% caolinite clay, 10% *sphagnum* peat) and pH was adjusted to 7.0 ± 0.5 by adding a saturated solution of calcium carbonate (OECD TG 207), which also acted as a control [9]. Loamy sand soil from the Turia River Natural Park (Valencia, Spain) was collected as the reference soil. It was taken from a non-agricultural area at a topsoil sampling depth of 0–20 cm. In order to avoid interference with ecotoxicity tests, we selected a reference soil that had not been treated with pesticides or fertilizers for at least 2 years. In the laboratory, the reference soil was sieved through a mesh (particle size 2 mm) and moisture was adjusted to 40% water holding capacity (WHC) with distilled water. For the carbon transformation test, 3000 g of soil on a dry weight (dw) basis were employed in each test sample. According to the TG, pre-incubation was carried out as bulk samples at 20 ± 2 °C under aerobic and dark conditions. The tests were conducted in triplicate.

#### 2.3.1. Carbon Mineralization Test

Substrate-induced respiration was measured as described in OECD TG 217 [39]. In this test, the respiration of any aerobic heterotrophic microorganisms present in soil was induced by adding glucose as a carbon source. The toxicity indicator is the glucose-induced respiration rate related to the control during incubation with problem soils. Three 1000 mL measuring MG OxiTop^®®^ vessels (WTW™, Weilheim, Germany) per treatment were filled with 100 g of the dry sieved soil or dilutions (four serial 1:2 mixtures from 50% to 6.25%) were prepared for all four samples. Controls were amended with 4.0 g/kg dw of soil. Vessels were incubated in the dark at 20 ± 1 °C for 24 h. The O_2_ consumed during respiration was measured during incubation by an OxiTop^®®^ Sensomat system (WTW™), which determines the pressure drop caused by consumed O_2_. To sequester the CO_2_ produced by respiration, a beaker with 50 mL of 1 M NaOH was allocated inside vessels.

#### 2.3.2. Nitrogen Mineralization Test

OECD TG 216 [40] was followed to investigate the long-term adverse effects of a substance on the N transformation activity of soil microorganisms. This bioassay studies the long-term effects of a test sample on the nitrifying activity of soil bacteria. Two measurements were taken: when the test started (0 day) and after 28 exposure days. A control (reference soil) and five serial 1:2 mixtures (50%, 25%, 12.5%, 6.25%, 3.12%) were prepared for all four samples. Aliquots of 500 g of dw soil per sample, adjusted to 50% of its WHC, were added to alimentary grade plastic (140 × 140 × 80 mm) containers. Each sample was amended with lucerne meal at a rate of 5 g/kg dw and was thoroughly mixed inside each container. After additions, samples and the control were incubated at 20 ± 2 °C for 28 days. Soil moisture was corrected by adding deionized water to 40–60% of the maximum WHC and was corrected weekly.

For N extraction, the triplicate 3 g soil aliquots were sampled from each container on day = 0 and day = 28 of incubation. Every 3 g of soil of each concentration were extracted with 20 mL of 0.5 M KCl/g dw in a shaker at 150 rpm for 1 h. Supernatants were centrifuged for 5 min at 6000 rpm and filtered using AP40 glass fiber 0.7 μm membranes (Millipore™). Filtrates were stored at −20 °C in 15 mL centrifuge tubes until the total NO_3_-N content measurement was taken by the Spectroquant^®®^ kit (No. 1.14773.0001) in a Nova60^®®^ spectrophotometer system (Merck Millipore^®®^, Darmstadt, Germany). The procedure was performed following the manufacturer’s instructions.

#### 2.3.3. Acute Toxicity Test with Earthworms

The test was based on OECD TG 207 [41] and conducted with the species *E. foetida.* Organisms were supplied by a local breeder (Lombritec^®®^, Guadalajara, Spain). In this test, only the clitellate adults of *E. foetida* weighing between 300 mg and 600 mg were selected. Earthworms were acclimated to the test conditions in the control soil at 20 ± 2 °C and fed with horse manure ad libitum for 5 days before testing started. Individuals were fasted to purge earthworm guts on the day before testing [4]. Ten organisms were placed in plastic alimentary grade containers (140 × 140 × 80 mm) containing 750 g dw of the test soil. The test containers were left in constant light (800 lx) at a temperature of 20 ± 2 °C. Survival was determined after 7 and 14 exposure days. Each test was run with four dilutions (50%, 25%, 12%, 6.25%), plus a control (only OECD soil) in duplicate per treatment. In parallel, weight loss was reported on days 0, 7 and 14 of exposure.

### 2.4. Aquatic Ecotoxicity Tests

Aquatic bioassays were performed using the soil water-leachable extracts obtained according to DIN 38414-S4 [14] during a batch test with a single leaching cycle (10 L/S) in a Reax20 rotary agitator (Heidolph™, Schwabach, Germany) for 24 h at room temperature. The solid and liquid phases were separated by sedimentation at 4 °C overnight. The leachate was separated by centrifugation at 3000× *g* for 10 min and filtered through a membrane (pore size 0.45 mm).

#### 2.4.1. Algal Growth Inhibition Test

Effects on microalgae growth were assessed according to the OECD TG 201 [42] and ISO 8692 [43] guidelines. Alginate beads of *Raphidocelis subcapitata* cells were supplied by Microbiotest Inc. (Ghent, Belgium). Algae were de-immobilized and left in constant light (white-tone LED lamps, 4000–5000 lx) at a temperature of 20 ± 2 °C. Five leachate dilutions per treatment (50%, 25%, 12.5%, 6.25%, 3.12%), plus a growth control that consisted in an algae culture medium, were run in triplicate. Wide-neck Erlenmeyer flasks (100 mL capacity) were filled with 75 mL of the test sample and inoculated with *R. subcapitata* to 10^4^ cell/mL. After 24 h, 48 h and 72 h of incubation, the absorbance of each replicate was measured at 670 nm in an Aurius™ 2021 spectrophotometer (CECIL Instruments, Cambridge, UK) using 100 mm optical glass cuvettes. To avoid interferences with spectrometric measures due to leachate coloration, specific blanks of each sample leachate were used. The results were indicated as a percentage of algal growth inhibition (% *I*) in relation to the control. The growth rate (*μ*) was calculated and the effect on algae growth inhibition was expressed as 72 h ErC_50_.

#### 2.4.2. Daphnia Magna Acute Immobilization Test

Assays were carried out in accordance with the conditions set by OECD TG 202 [44] and ISO 6341 [45]. The commercial kit Daphtoxkit F^™^ (Microbiotests Inc., Gent, Belgium) was used to perform them. The followed procedure is summarized in the Standard Operating Procedure supplied by the manufacturer [46]. Acute toxicity was assessed by noting the effects of the test leachates on *D. magna* motility. The daphnids that underwent hatching for less than 24 h were obtained from dormant eggs (ephippia). Two hours before tests began, neonates were fed a suspension of *Spirulina* sp. powder. Tests were conducted in the dark at 21 ± 1 °C. Organisms were considered immobile if they had settled at the bottom of the test container 48 h after incubation with the toxicant and did not resume swimming during the 15 s observation period. Daphnids were exposed to six dilutions of aqueous extracts (100%, 50%, 25%, 12.5%, 6.25%, 3.12%) (*v*/*v*), plus a control in four replicates per treatment. EC_50_ was determined as the dilution (%) required to immobilize 50% of daphnids after an exposure time from 24 h to 48 h.

### 2.5. Statistical Analysis

The descriptive statistics were computed for the PTE concentrations for soils and leachates. Toxicity was expressed as the percentage of effect and, whenever possible, as the median effective concentration (EC_50_), along with 95% confidence limit values, as determined by the probit regression implemented in the EPA Probit software (v1.5). A one-way ANOVA was carried out, followed by Tukey’s post hoc analysis. The statistical analysis was done using SPSS Statistics™ (v21 for MS Windows™, IBM, Armonk, NY, USA).

## 3. Results

### 3.1. Soil Properties and PTEs Contents

Table 1 includes the physico-chemical properties of the studied topsoils and the summary statistics of the soil parameters. Soils generally have a medium-fine texture and a basic pH (7.55–8.01), and may present salinity problems (EC 0.78–1.68 dS/m), high carbonates contents, as well as a high SOM content for agricultural soils. Soil pH values are associated with high carbonate contents, which are typical of Mediterranean soils with more than 15% CaCO_3_ developed on calcareous materials. In general, the EC, SOM, N and CEC values were significantly lower in S1 and S2 compared to S3 and S4. The soils with higher SOM contents have higher CEC and N. This result is expected because the direct association between N and CEC with SOM is well known. The high SOM content in S3 and S4 may be due to urban wastewater inputs, a fact that has already been highlighted in previous studies [20,28]. Soils with high contents of clay, SOM and carbonates, and a basic pH, give way to low mobility of heavy metals in the soil solution, accumulate on the soil surface and are leached in very small quantities to lower soil horizons [7,38,47,48,49]

The risk of exposure of soil organisms to potentially toxic elements present in soil is related to the soil bioavailability of these elements [50]; EDTA is a widely used chelating compound in soils as an indicator of the mobility and bioavailability of PTEs and micronutrients [7,38,51]. Jalali et al. [52] concluded that EDTA was the best reagent for the extraction of most PTEs compared to other extracting solutions like CaCl_2_, DTPA, HNO_3_, MgCl_2_, NaNO_3_, NH_4_NO_3_ and NH_4_OAc. NaNO3 is, therefore, a suitable extractant to indicate bioavailability compared to other compounds.

The study of Co, Cr, Cu, Ni, Pb and Zn content in the EDTA-extractable fraction in the sampled soils (Table 2) showed that the content of these elements in S3 and S4 was significantly higher (*p* < 0.05) than in S1 and S2, which indicated the major availability of these metals in S3 and S4. These results, along with the high EC, SOM and N values, are a clear indicator of the high degree of anthropization to the north of the ANP (S3 and S4) versus its southern part, namely, S1 and S2. Boluda et al. [20] determined the content of pseudo-total heavy metals in soil samples from rice fields to the north and south of the ANP, and obtained a higher concentration in the north than in the south, which was corroborated by our results. This distribution of pollutants in the paddy soil of ANP has also been observed with some organic contaminants. The high concentration of pesticides in soil [29], and drugs and pharmaceutical in the waters [53] and sediment [22] of the irrigation channels used near paddy fields, were found in the northern area (S3 and S4) compared to the lower concentration encountered to the south of the ANP. The above-cited results, along with those herein obtained, stress higher multipollution by organic and inorganic contaminants that present the different matrices (water, soil and sediment) obtained to the north of the ANP, which would be related to higher population density, spillage of water from wastewater treatment plants (WWTPs) and discharges from industrial and urban wastewater to the north of the ANP [22,28,29,53]. Iranzo et al. [54] observed the significant presence of pharmaceuticals in the water and sludge from WWTPs that discharge their water into the irrigation channels of the rice fields in the ANP.

The available concentrations of all the potentially toxic elements obtained in S3 and S4 were higher than the mean values obtained in greenhouse soils [7,38], on farmlands where horticultural crops are grown [11,56], and in urban soils of the Seville city (South Spain) [55]. The S1 and S2 samples did not generally present higher PTEs values than those obtained in the studies indicated in Table 2. Soils with ZnEq values higher than 17 mg/kg are considered to indicate toxicity [38], which is the case of S3 and S4. The EC, nitrate content, Ca, K, Cu, Na and P concentrations were slightly higher in the more anthropized northern area. Generally, the concentrations of most of the elements analyzed in aqueous extracts were below 0.01 mg/L. The Cu, Ni, Pb and Zn concentrations in the aqueous leachate were much lower than the available concentrations present in soils. These facts demonstrate the low mobility of such elements in these soils, which has also been shown to be the case in contaminated soils from mining activities [4]. The properties (a basic reaction, fine texture, strongly calcareous, a high SOM, CEC and base saturation) of the soils used for growing rice in the ANP give way to poor heavy metals bioavailability because they can be strongly fixed to the solid soil phase in different forms, such as: clay minerals, organic matter or co-recipitates like carbonates or sulfides, and Fe and Al oxides and hydroxides. Therefore, they do not move to the aqueous leachate [20].

### 3.2. Soil Ecotoxicity Assessment

The applied ecotoxicity tests were not sufficiently sensitive to determine EC_50_ in the studied soils (Table 3) despite the soils from Alfafar and Massanasa presenting higher available heavy metals values than those reported in other studies [7,38,55], whose soil samples came from land uses with a potential pollution risk caused by these elements. For example, mining soils with high heavy metals contents that were included in the assays done with *E. foetida* (OECD TG 207) were not sensitive enough to estimate EC_50_s [4]. Soils from mining areas in Portugal showed no earthworm mortality [57]. To a certain extent, inhibition was observed in nitrifying activity (OECD TG 216) and algal growth (OECD TG 201) in soil S4 with EC_50_ (%) values of 98.0 and 68.6, respectively. This result indicates that these tests could be the most sensitive ones for the available PTEs concentration and properties in this soil. Others studies have performed the soil respiration test (OECD TG 217) and applied organic amendments or used soils with high organic matter contents but detected no toxic effects, not even at high metal concentrations [19].

#### 3.2.1. Terrestrial Ecotoxicological Bioassays

**Figure 2:**
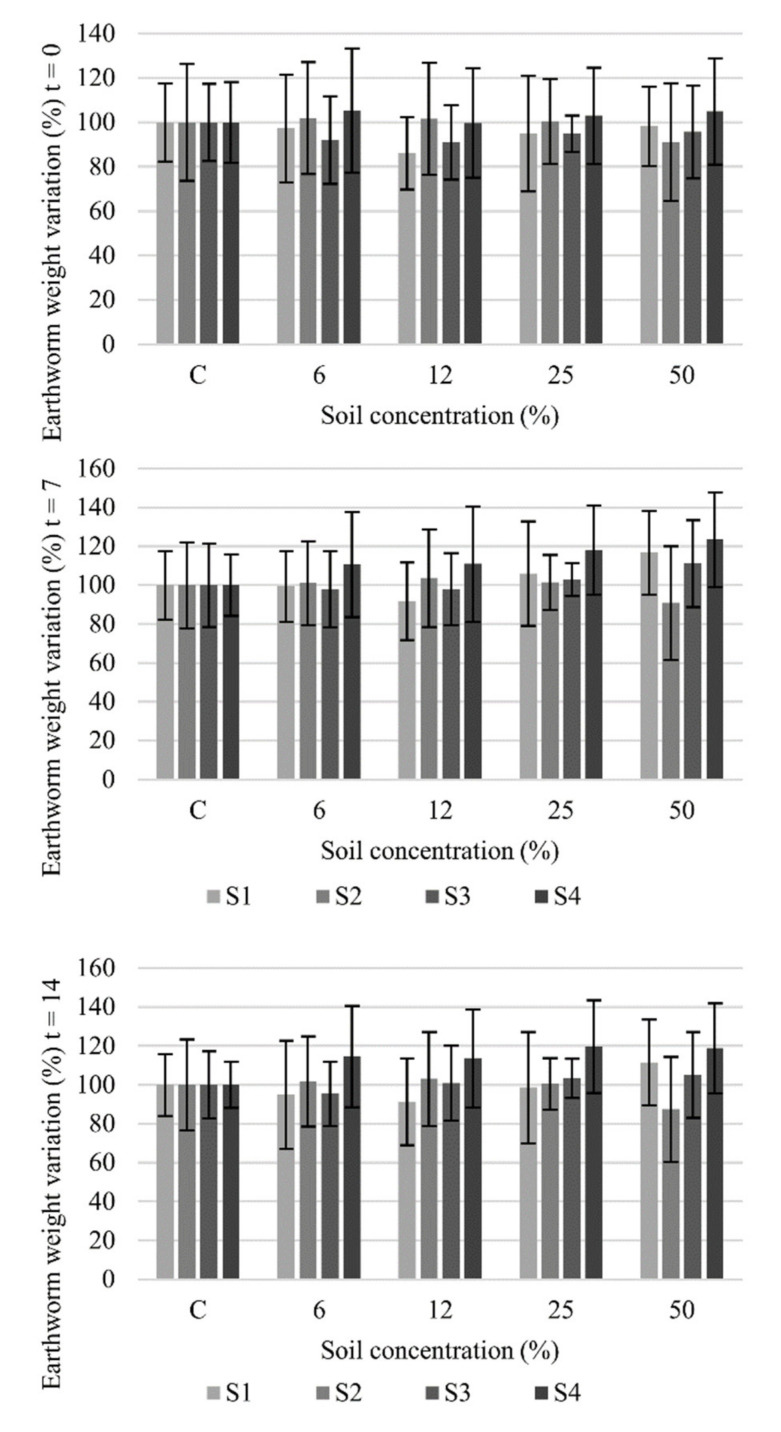
Earthworm weight variation (%) at t = 0, 7 and 14 days (mean ± SD, n = 10) for each soil concentration and control (C) in the towns of Sueca (S1), Sollana (S2), Massanassa (S3) and Alfafar (S4).

The *E. foetida* earthworm assay showed no toxic effect because no mortality was found at any of the tested concentrations. Likewise, no statistically significant variations (Tukey’s, *p* < 0.05) were noted in the weight of the exposed earthworms compared to the controls. Earthworms did not show any behavioral changes (e.g., to avoid substrate) while testing. Earthworm weight dropped weekly for all the controls. Except for S2, which displayed the same biomass loss tendency as the controls, the biomass of the other samples increased after a given dose in the first week, and approximately returned to the initial biomass after 14 days (Figure 2). This effect might be due to the fact that when the soil dose was increased, the SOM in the mixture with artificial soils also rose. The organic matter present in soil was used as a food source for earthworms, which was eaten in approximately 7 days. The mass loss observed in the controls began after this initial period. Alvarenga et al. [9] reported similar results for their studies done in soils with more trace elements present. Hence the presence of metals at subchronic concentrations, as in our case, could diminish earthworm activity by giving way to less mobility and, thus, by reducing intake.

Greater biomass increase took place in 50% of the mixture in sample S4. SOM content was higher, but this effect was not observed in S2, which coincided with the sample with lower SOM. Therefore, earthworms may accumulate metals through the intake of the soil components linked with metals or directly through the dermal absorption of dissolved ions. Nevertheless, earthworms have the capacity to regulate and excrete them. This effect is stressed with Zn, Pb and Cu (presence of these elements was greater in the analyses) that, along with low bioavailability, limits absorption or intake of this species, which could avoid harm to this species [58,59].

**Figure 3:**
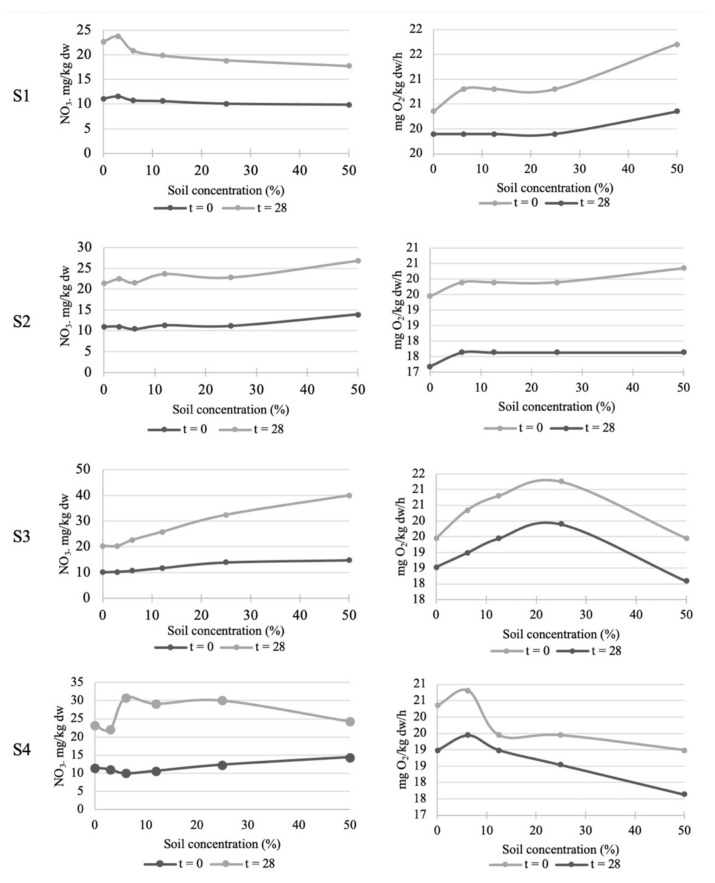
Variation in nitrates concentration and O_2_ consumption between t = 0 and 28 days for each soil dose in the towns of Sueca (**S1**), Sollana (**S2**), Massanassa (**S3**) and Alfafar (**S4**).

Figure 3 shows variation in the consumed O_2_ and nitrate concentration at different times and doses in the studied soils. As previously mentioned, although the N and C mineralization tests showed no toxicity, some variation was noted in the measurement time and the applied soil dose. Generally, for all the studied doses and soils, a reduction in O_2_ consumed was evidenced at t = 28 days compared to the measurements taken at the zero time point (t = 0). These results are similar to those obtained by Carabassa et al. [19], which showed diminished relative respiration after 28 days in Cu-polluted soils, and to that reported by Ritz et al. [60], who revealed that substrate-induced respiration increased only on the first 25 days of incubation. For samples S1 and S2, the respiration rate increased compared to the control (0% concentration) and tended to increase with a higher concentration of both these soils. This would stimulate biological activity by organic matter content increasing as the soil dose grew higher. In line with this, it has been verified that the respiration rate of soils increases with higher organic matter content, among other variables [61]. Respiration in soil samples S3 and S4 gradually increased with higher soil doses up to 25% and 6.25%, respectively, and then decreased at times t = 0 and t = 28. These results might indicate hormesis to a certain extent, as pointed out by studies conducted in soils with different doses of heavy metals and organisms [62,63]. The effect of high heavy metals concentrations on soils tends to be negative on respiration [2]. Therefore, mining lands with high heavy metals contents have shown a lower respiration rate than farmland where these contents are lower [64]. Moreover, one study added different Cu, Zn and Cr doses to soil and found that high concentrations had a negative effect on substrate-induced respiration [19].

The N mineralization test (Figure 3) revealed that after 28 incubation days, the nitrates concentration in all the soils and for all the added soil doses was higher than when testing began (t = 0). This result would be related to the greater organic matter mineralization observed after 28 incubation days. In S2 and S3, the nitrates concentration increased with the soil dose to be tested at both t = 0 and t = 28, which could be due to a higher initial nitrates content because these soils are used for agricultural purposes. Samples S3 and S4 had a higher nitrates content after 28 incubation days compared to S1 and S2, possibly because the higher SOM and N values in these samples could favor nitrate formation. However, it is worth highlighting: as of the 6.24% dose for S4, nitrifying activity began to gradually lower until values were below those obtained for the controls at the 50% dose after 28 days; at a higher heavy metals concentration for S4, the concentration of this soil increased, which could affect nitrifying organisms in relation to the reference soil. In general terms, the microorganisms involved with N transformation are inhibited directly or indirectly by heavy metals, depending on not only the concentration and oxidation state of heavy metals, but also on soil characteristics [18], which is why the Cd contamination of paddy soils decreases the N transformation process [17].

#### 3.2.2. Aquatic Ecotoxicological Bioassays

The *D. magna* assay detected no toxicity at any studied dose, organisms displayed no abnormal behaviors, erratic movements or immobilization after 48 h at any dose, and the percentage of immobilization in the undiluted leachates (whole elutriate) was 0%. These results might be explained by the low concentration of the elements present in leachates. Some studies [4,57,65] have been performed with soil leachates. They indicate a higher concentration of heavy metals and metalloids (Co, Cu, Zn, As, etc.), and found no high toxicity in the assays run with this organism. Cui et al. [66] compared sensitivity to heavy metals in *D. magna* and *D. galeata* by adding different concentrations of several metals to their respective environments. In this regard, *D. galeata* was more sensitive to Cr, Fe, Ni and Pb, and both species were similarly sensitive to Cu, Cd and Zn. In any case, the levels of these metals employed to evidence toxicity were much higher than those found in the leachates of the present study. However, it is worth bearing in mind that assays were done individually with metals, which eliminates any possible interactions or synergies among different pollutants.

The *R. subcapitata* growth inhibition test only detected a minor inhibitory effect on the 50% dilution with sample S4. All the other samples displayed a hormetic effect for stimulation on growth. The growth rate inhibitory effects (*I*%) for each sample site are shown in Table S1, where negative values denote a stimulation effect on algae growth. This stimulation effect (known as hormesis) in algae has been described in many studies conducted with different substances, such as metal oxide-based, environmental samples, nanoparticles and pesticides [67,68,69,70]. The fact that algal density lowered might suggest that certain pollutants started to interfere with microalgal development. This drop in S1 and S2 was not very pronounced. In S3 and S4 for the 50% dose, this reduction was lower than the control and could be due to the higher pollution level in these two samples. The curve fitting values for Probit regression are shown in Table S2.

The Cu and Mn contents in leachate were higher than 0.02 mg/L, but this did not cause an effect on the tests done with *D. magna*. In the assays with *R.* *subcapitata,* slight stimulation was noted in the growth rate (72 h ErC_50_ > 100%) compared to the control (Figure S1). The presence of supplementary macronutrients in soil extracts (S, Mg, Na, Ca, K, P, Co, Zn) even below the quantification limits (0.01 mg/L) could explain this stimulatory effect. According to OECD TG 201 (Annex 3) [42], many of them (including Cu and Mn) are used to prepare algal growth media. The Cu concentration in the aqueous extracts was below both 72 h ErC_50_ = 30–824 μg Cu/L for *R. subcapitata* according to De Schamphelaere and Janssen [71], and 48 h EC_50_ 213–438 μg Cu/L calculated for *D. magna* in accordance with the studies by De Schamphelaere et al. [72].

According to the European Chemicals Agency [73], Mn has a 72 h ErC_10_ = 3.4 mg Mn/L for algae and a 48 h EC_50_ = 0.65 mg Mn/L for *D. magna*. Although the Mn concentrations were higher than the EC_50_ values for algae and *D. magna* in all the water extracts, neither inhibition growth in algae nor mortality effect in daphnids was detected.

For all the above reasons, although ecotoxicity methods using soil extracts simulate the mobility of these PTEs under natural conditions, they are inefficient for evaluating toxicity due to these elements, and according to the properties and nature of the soils selected for this study, because soil aqueous extracts do not reflect the available concentration of PTEs. Therefore, ecotoxicity methods that put the target organisms in contact with the soil matrix are likely to be more efficient [20].

## 4. Conclusions

The stronger anthropic pressure noted to the north of the ANP (due to high population density, industrial activity and the discharge of wastewater into irrigation channels) gives way to a higher concentration of available PTEs in the paddy fields of Massanassa (S3) and Alfafar (S4) than in Sueca (S1) and Sollana (S2) in the south. The higher assimilable concentration of PTEs in S3 and S4 vs. S1 and S2 was not observed in water-leachable extracts due to the physico-chemical characteristics and nature of the studied soils. Therefore, the water extraction procedure (DIN 38414-S4) does not seem to be efficient enough to extract all the trace metals present in whole soils, but it is required by regulations for being compatible with the application of aquatic ecotoxicity bioassays.

Applying a battery of ecotoxicological bioassays combined with the physico-chemical and edaphological characterization of the studied soils is a useful tool for an environmental risk assessment. Although the soils in the northern zone showed high levels of available PTEs, the ecotoxicological bioassays revealed no toxicity and gave an EC50 >1% in all the bioassays, which are considered “non-polluted” soils. The low sensitivity of the acute toxicity assays seemed related to the physico-chemical characteristics of the studied soils (basic pH, high SOM content, clay texture) and not only the bioavailable heavy metals levels. Hence, there is a need to update the test battery by including bioassays that integrate behavioral endpoints, such as feeding or reproductive inhibition and avoidance habitat.

## Figures and Tables

**Table 1:** Physico-chemical properties of the soil samples (mean ± standard deviation, n = 2) and soil leachates.

Soil										Leachates		
Samples	Sand	Silt	Clay	pH	EC	CaCO_3_	SOM	N	CEC	pH	EC	NO_3__−_
	%				dS/m	%	%	%	cmolc/kg		dS/m	mg/L
S1	6 ± 2 b	53 ± 1 a	41 ± 2 a	7.92 ± 0.12 a	0.78 ± 0.07 a	35.96 ± 1.93 a	5.29 ± 0.09 bc	0.32 ± 0.01 c	23.69 ± 2.01 ab	8.10	0.72	9.60
S2	7 ± 1 b	50 ± 1 a	44 ± 1 a	8.01 ± 0.10 a	0.50 ± 0.06 a	35.52 ± 1.25 a	4.39 ± 0.05 c	0.25 ± 0.01 c	18.90 ± 1.20 b	7.96	0.53	8.83
S3	23 ± 1 a	49 ± 1 a	28 ± 1 b	7.70 ± 0.08 b	1.22 ± 0.11 bc	34.11 ± 1.02 a	7.01 ± 0.09 b	0.41 ± 0.01 b	27.4 ± 1.82 ab	8.01	0.73	10.26
S4	26 ± 1 a	53 ± 1 a	21 ± 1 b	7.55 ± 0.15 c	1.68 ± 0.18 c	30.09 ± 2.01 a	8.89 ± 0.11 a	0.52 ± 0.02 a	30.8 ± 2.56 a	7.75	0.60	10.16

Significant differences among the four soils (*p* < 0.05) are indicated by different letters after the ANOVA by Tukey’s post hoc analysis. EC: electrical conductivity; SOM: soil organic matter; CEC: cation exchange capacity.

**Table 2:** Available (EDTA) metals concentration (mg/kg) and zinc equivalents in the sample soils of different Spanish soils (Mean ± standard deviation, n = 2); total element concentrations (mg/L) in the tested soil leachates.

Samples and Reference Soils	Soil						
	Co	Cr	Cu	Ni	Pb	Zn	Eq. Zn
S1	0.10 ± 0.02 b	0.18 ± 0.01 b	5.14 ± 0.40 c	0.32 ± 0.02 b	4.75 ± 0.51 b	1.55 ± 0.23 b	14.36
S2	0.27 ± 0.03 ab	0.22 ± 0.01 b	5.01 ± 0.62 c	0.51 ± 0.04 b	2.74 ± 0.35 b	2.06 ± 0.31 b	16.18
S3	0.40 ± 0.03 a	0.60 ± 0.04 a	42.20 ± 3.50 b	8.40 ± 0.70 a	47.50 ± 5.04 a	57.03 ± 5.71 b	208.60
S4	0.30 ± 0.04 a	0.80 ± 0.08 a	71.30 ± 8.10 a	8.90 ± 0.94 a	61.80 ± 7.20 a	179.15 ± 18.90 a	392.95
Gil et al. [38]	1.6	-	6.8	1.7	24.3	11.5	-
Ramos-Miras et al. [7]	1.7	-	9.5	1.7	26.3	11.8	44.6
Madrid et al. [55]	-	3.73	18.4	0.57	65.4	35.5	-
Peris et al. [11]	0.23	0.07	6.5	0.54	20	18.3	-
	**Leachates**						
	Ca/Mg	K/P	Na/S	Si/Fe	Mn	B	Cu
S1	112.93/20.64	6.37/0.22	44.19/82.5	3.99/0.02	20.64	0.12	0.04
S2	75.25/12.46	2.40/0.11	36.86/38.30	2.02/0.02	12.46	0.04	0.03
S3	94.96/15.80	8.48/0.91	53.84/75.75	3.68/0.02	15.80	0.29	0.09
S4	76.38/12.97	6.94/0.32	43.31/50.83	3.21/0.02	12.97	0.13	0.04

Significant differences among the four soils (*p* < 0.05) are indicated by different letters after the ANOVA by Tukey’s post hoc analysis.

**Table 3:** EC_50_ of the ecotoxicological tests. Values expressed as % of soil/leachate in the dilution (*w*/*w, v*/*v*). Soil classification according to Spanish Royal Decree 9/2005. NC: “Not Contaminated” (EC_50_ > 1%).

Contact	Organism	Endpoint	Ecotoxicolog-ical Parameter	Soil Sample			
				S1	S2	S3	S4
Direct (whole soil)	*Eisenia foetida*	Mortality	14 d EC_50_ (%)	>50%	>50%	>50%	>50%
	Soil microorganisms (N_2_)	Nitrogen transformation	28 d EC_50_ (%)	>50%	>50%	>50%	98%
	Soil microorganisms (O_2_)	Carbon transformation	28 d EC_50_ (%)	>50%	>50%	>50%	>50%
Indirect (soil leachate)	*Daphnia magna*	Immobilization	48 h EC_50_ (%)	NT	NT	NT	NT
	*Raphidocelis subcapitata*	Growth rate	72 h ErC_50_ (%)	>50% (102 *)	>50% (105 *)	>50% (110 *)	68.6%
Soil classification				NC	NC	NC	NC

NT: Non-toxic (no mortality in the undiluted leachate). (*) EC_50_ value calculated by Probit regression.

## Data Availability

Oscar Andreu-Sánchez, PhD, as responsible of the manuscript entitled “Application of an Ecotoxicological Battery Test to the Paddy Field Soils of the Albufera Natural Park”, on behalf of the rest of the coauthors, with this document I warrantee and sign that the datasets generated and used during the current study are available from the corresponding author on reasonable request.

## Supplementary Materials

The following supporting information can be downloaded at: https://www.mdpi.com/article/10.3390/toxics10070375/s1. Table S1: Growth rate inhibitory effect (*I*%) obtained in the algal test with *R. subcapitata*. Table S2: Probit curve fitting values for the *R. subcapitata* assays, Figure S1: Probit regression curves for algal assay, Figure S2: Experimental photographs of the bioassays performed.
