# Supplementary material for: Application of an Ecotoxicological Battery Test to the Paddy Field Soils of the Albufera Natural Park
Source: Toxics. 2022 Jul 5;10(7):375. doi: 10.3390/toxics10070375 (PMC9324136; doi:10.3390/toxics10070375)
Supplement: Supplementary file 1 [file toxics-10-00375-s001.zip › toxics-1748620-supplementary.pdf]

toxics

Supplementary Materials: Application of an Ecotoxicological Battery Test to the Paddy Field Soils of the Albufera Natural Park

Oscar Andreu Sánchez, Jesús Moratalla López, José Antonio Rodríguez Martín and Luis Roca Pérez

Table S1. Growth rate inhibitory effect (*I*%).

Sample Sites	Exposure Concentration (% of leachate)				
	3.12%	6.25%	12.5%	25%	50%
S1	-0.0134	-0.0039	0.0032	0.0041	0.0890
S2	-0.0119	-0.0053	0.0073	0.0052	0.0903
S3	-0.0140	-0.0046	0.0093	0.0081	0.0981
S4	-0.0012	-0.0100	0.0210	0.1210	0.3732

Negative values denote growth stimulation.

Table S2. Probit curve fitting values.

Data	Sample Sites			
	S1	S2	S3	S4
Intercept				
Value	−3.7632	−3.3845	−2.73038	0.26296
Std. Err.	0.96018	0.87548	0.7201	0.5620
C.I.(95%)	−5.64522 /−1.88129	−5.1005 / −1.66862	−4.14179 / 1.318964	−0.8385 / 1.3645
Slope				
Value	4.3654	0.875483	3.79016	2.5771
Std. Err.	0.5745	0.52537	0.434970	0.3711
C.I.(95%)	3.2393 / 5.4916	3.1167 / 5.1761	2.9376 / 4.6427	1.8497 / 3.3044

Std. Err: Standard Error. CI95%: Confidence Intervals (95%).

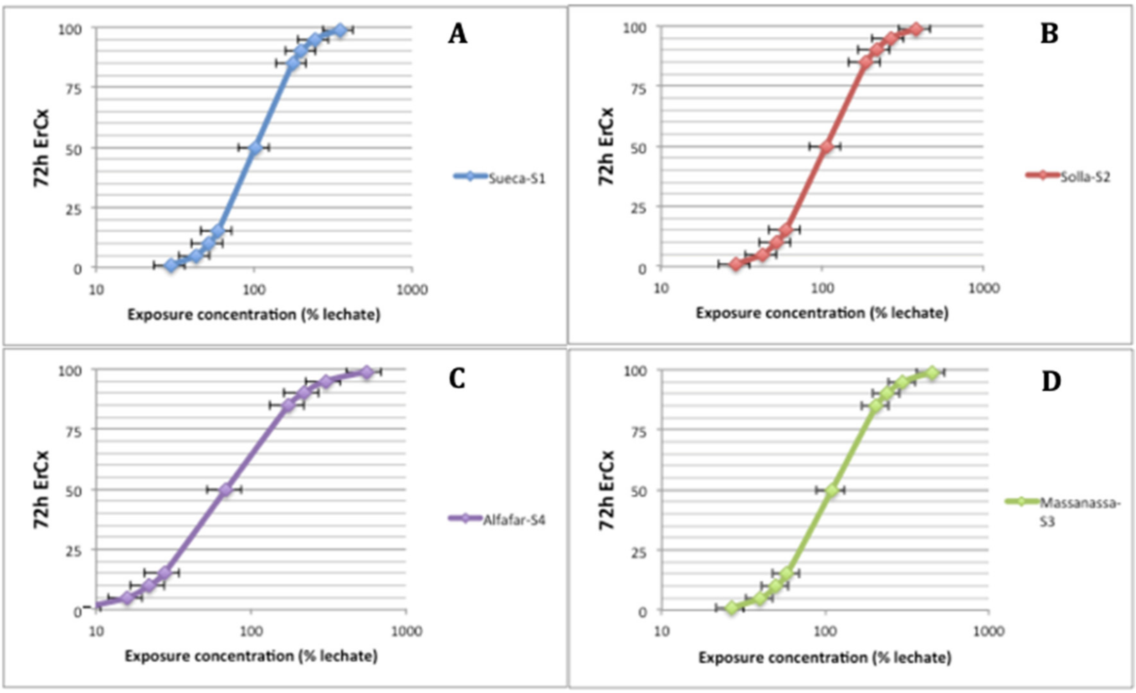

Figure S1. Probit regression curves for the *R. subcapitata* assay. X-axis: exposure concentration expressed as % of leachate. Y-axis: 72h ErCx. Error bars denote the interval of confidence (95%). Sample sites: A) S1, B) S2, C) S3 and D) S4.

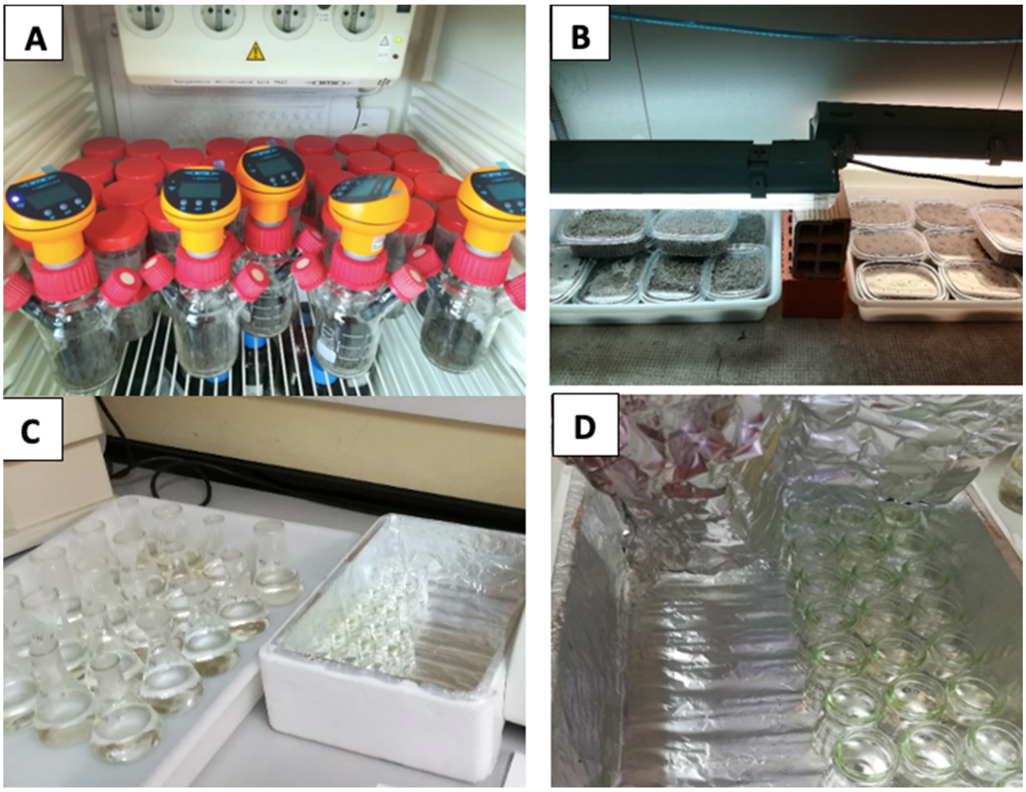

Figure S2. Experimental photography. (**A**) Respirometry bottles with Oxitop™ system for carbon fixation assay (OECD TG 216). (**B**) Plastic containers for the earthworms mortality assay (OECD TG 207). (**C**) Erlenmeyer flasks with different contraptions of leachate inoculated with *R. subcapitata* (OECD TG 201). (**D**) Trays with rinsing vials for *D. magna* assay (OECD TG 202).
